# Peroxynitrite Sensor Based on a Screen Printed Carbon Electrode Modified with a Poly(2,6-dihydroxynaphthalene) Film

**DOI:** 10.3390/s16111975

**Published:** 2016-11-23

**Authors:** Ioana Silvia Hosu, Diana Constantinescu-Aruxandei, Maria-Luiza Jecu, Florin Oancea, Mihaela Badea Doni

**Affiliations:** National Institute for Research & Development in Chemistry and Petrochemistry—ICECHIM, Biotechnology Department, 202 Spl. Independentei, Sector 6, Bucharest, Romania; ioanasilvia.hosu@ed.univ-lille1.fr (I.S.H.); diana.constantinescu@icechim-rezultate.ro (D.C.-A.); jecu.luiza@icechim.ro (M.-L.J.); dir.stiintific@icechim.ro (F.O.)

**Keywords:** 2,6-dihydroxynaphthalene, electropolymerization, screen printed carbon electrode, peroxynitrite, cyclic voltammetry, amperometry, flow injection analysis, electrocatalysis

## Abstract

For the first time the electropolymerization of 2,6-dihydroxynaphthalene (2,6-DHN) on a screen printed carbon electrode (SPCE) was investigated and evaluated for peroxynitrite (PON) detection. Cyclic voltammetry was used to electrodeposit the poly(2,6-DHN) on the carbon electrode surface. The surface morphology and structure of poly(2,6-DHN) film were investigated by SEM and FTIR analysis, and the electrochemical features by cyclic voltammetry. The poly(2,6-DHN)/SPCE sensor showed excellent electrocatalytic activity for PON oxidation in alkaline solutions at very low potentials (0–100 mV vs. Ag/AgCl pseudoreference). An amperometric FIA (flow injection analysis) system based on the developed sensor was optimized for PON measurements and a linear concentration range from 2 to 300 μM PON, with a LOD of 0.2 μM, was achieved. The optimized sensor inserted in the FIA system exhibited good sensitivity (4.12 nA·μM^−1^), selectivity, stability and intra-/inter-electrode reproducibility for PON determination.

## 1. Introduction

Peroxynitrite (ONOO¯), a very powerful oxidizing agent, is formed in vivo from the nearly diffusion-limited termination reaction between nitric oxide (NO^·^¯) and superoxide (O_2_^·^¯). The coexistence of peroxynitrite (PON) precursors in biological tissues used for food, especially in skeletal muscles, implies the PON generation. Since it was reported that peroxynitrite enhances the lipid oxidation [1] and promotes the conversion of oxymyoglobin to metmyoglobin in skeletal muscle [2,3], the PON monitoring and control in the early stages of processing may be important to delay the oxidation processes that affect the quality of meat and meat based products [4,5].

Various methods were developed to detect peroxynitrite. The fluorescent probes method, usually using dihydrodichlorofluorescein (DCFH) and dihydrorhodamine-123 (DHR-123) as the probes [6,7], has been widely employed in the mid-90 s to monitor peroxynitrite in various biological systems. Lately, new fluorescent probes such as rhodamine B hydrazide [8], HKGreen-1 [9], folic acid [10], boronates [11,12,13], rhodol [14] for PON detection were described. Very recently, the advances in carbon dots synthesis allowed the development of florescent probes for PON sensing based on carbon dots obtained from tryptophan-doped glucose [15] or from citric acid and urea [16]. Methods based on chemiluminescence [17], UV-Vis spectroscopy [18,19], EPR spectroscopy [20] were also reported. The abovementioned methods are indirect methods based on measurements of secondary species and they lack sometimes the sufficient specificity needed for PON detection in biological samples.

During the last decade electrochemistry has become very attractive for sensing of important reactive species present in biological samples due to the possibility of direct, on-line and real-time measurements [21,22,23]. Amatore et al. [24] studied for the first time the unmediated electrochemical behavior of PON on Pt disk microelectrodes. One of the first electrochemical methods for PON detection in simulated bodily fluid at a bare platinum electrode by linear scan voltammetry was reported by Iwuntze et al. [25]. The process involved the two-electron oxidation of peroxynitrite and a detection limit of 1.6 × 10^−7^ M was reported. Afterwards, different chemical modifiers were used to modify Pt electrodes. The first one was an electropolymerized manganese tetraaminophthalocyanine film (MnTAPc) used for the determination of PON in alkaline aqueous solution. Bedioui’s group showed for the first time a real-time calibration curve of the amperometric determination of stable PON in aqueous solution. MnTAPc film deposited on Pt microelectrodes presented electrocatalytic features on reduction of PON at −0.5V vs. Ag/AgCl. The sensitivity of the sensor was 14.6 × 10^−6^ μA·μM^−1^ and its detection limit is 5 × 10^3^ μM PON [26]. A PON sensor (wrongly defined as biosensor) has been developed through the preparation of a manganese-[poly-2,5-di-(2-thienyl)-1*H*-pyrrole)-1-(*p*-benzoic acid)](Mn-pDPB) complex. DPB monomer synthesis and polymerization for the purpose of providing a polymer backbone for complex formation with Mn^2+^ ion was for the first time reported by Koh et al. [27]. This report claiming that the Mn-pDPB based sensor allows the electroreduction of PON at +0.2 V with a sensitivity of 0.157 ± 0.007 μA·μM^−1^ has risen some comments with respect to certain issues such as the neutral pH of the analyzed PON solutions due to the very short half-life of PON at physiological pH [28]. Questionable was also the nature of the PON. Koh responded partially to this questions arguing that PON was introduced via a donor solution of 3-morpholinosydnonimine (SIN-1) which is relatively stable at neutral pH, having a half-life of 14–26 min [29].

A glassy carbon electrode (GCE) modified with electropolymerized film of cyanocobalamin to catalyze the peroxynitrite oxidation at a potential around +0.75 V was reported by Wang and Chen [30]. PON was sensitively detected at pH 9.2 by differential pulse voltammetry with the developed poly(cyanocobalamin) sensor in the range 2–300 μM with a detection limit of 0.1 μM. Since the electroactive polymers such as polypyrrole, polyaniline or poly(3,4-ethylenedioxythiophene) (called PEDOT) become very attractive for electrochemical sensors development [31], a PON sensor using carbon fiber microelectrodes modified with functionalized PEDOT-hemin was reported for the first time by Peteu et al. [32]. These PEDOT-hemin based sensors allowed the electrocatalytic oxidation of PON at +0.75 V vs. Ag/AgCl, in pH 10.5 CAPS buffer solution with a 5 s response time, 0.2 μM limit of detection and an excellent sensitivity of 0.013 μA·μM^−1^ [32]. The intrinsic catalytic role of hemin electropolymerized film on carbon electrodes in oxidative detection of PON was also investigated by Peteu et al. [33]. The modified electrode showed an oxidation peak at about +1.07 V vs. Ag/AgCl, in the presence of PON in pH 10.5 buffer solution. In amperometry, at potential of +0.7 V vs. Ag/AgCl , the response of the hemin carbon fiber electrodes was at least four times larger than the bare carbon microelectrode [33]. Based on the results showing improved electrochemical response for some electroactive species at conducting interfaces modified with reduced graphene oxide (rGO) a promising rGO/hemin modifed glassy carbon interface for the sensitive electrocatalytic detection of peroxynitrite at neutral pH was proposed [34]. The rGO/ hemin matrix was formed by a facile and environmentally friendly approach based on the reduction of GO with hemin under ultrasonication at room temperature. The rGO/hemin sensor showed in amperometry, at E = +1.1 V vs. Ag/AgCl, a very good sensitivity (7.5 ± 1.5 μA·μM^−1^) and low detection limit (5 × 10^−3^ μM) for PON generated by SIN-1 [34]. Recently, the electrocatalytic activity of cobalt phthalocyanine tetracarboxylic acid (CoPc–COOH) loaded on reduced graphene oxide (rGO) films towards PON was investigated [35]. The developed sensor proved to be also very sensitive (11.5 ± 1 μA·μM^−1^) in chronoamperometry at the potential E = +1.1 V vs. Ag/AgCl. Very recently the Peteu’s group reported a hemin-PEDOT functionalized boron-doped diamond microelectrode for PON detection with LOD = 0.01 μM [36].

Almost all of the reported electrochemical sensors based on the electrocatalytic oxidation of PON are operating at a voltage higher than +0.7 V where many other compounds present in the biological samples like meat food are susceptible to electrochemical oxidation. In this work we investigate the electrocatalytic features towards PON of the poly(2,6-dihydroxynaphthalene). The first sensor assembled by the 2,6-dihydroxynaphthalene (2,6-DHN) electro-copolymerisation with 2-(4-aminophenyl)-ethylamine (AP-EA) was done onto Pt electrodes [37]. The resulted film proved to be a very good support for enzyme immobilization with a remarkable rejection of interferences such as ascorbic acid or acetaminophen. Poly(2,6-DHN) electrodeposited on Pt was reported as a very selective amperometric sensor for nitrite determination (E = +0.9V) in meat extracts with pH 4.0 [38]. Herein, the authors observed that poly(2,6-DHN) electropolymerised on carbon electrodes shows catalytic properties on electrooxidation of PON in alkaline solutions at potentials much lower (~+0.1 V) comparing with the previous mentioned examples. In this paper we investigated the quantification of PON with a cost-effective sensor based on poly(2,6-DHN) electropolymerised on a screen printed carbon electrode (SPCE).

## 2. Materials and Methods

### 2.1. Chemicals and Materials

All chemicals, i.e., 2,6-dihydroxynaphthalene (Aldrich, Steinheim, Germany), Na_2_HPO_4_·2H_2_O (Riedel-de Haën, Seelze, Germany), NaH_2_PO_4_·2 H_2_O (Merck), KCl (Sigma-Aldrich, Darmstadt, Germany), NaOH (Sigma, Darmstadt, Germany), NaNO_2_ (Riedel-de Haën, Seelze, Germany), H_2_O_2_ 30% (Sigma-Aldrich), MnO_2_ (Merck, Darmstadt, Germany), HCl (Merck) and ascorbic acid (Sigma-Aldrich) were used without any further purification. Bidistilled water was used to prepare all solutions. Screen printed carbon electrodes (SPCE, Product code: DRP-110) from DropSens, (Llanera, Asturias, Spain) were used in all electrochemical experiments.

### 2.2. Electrochemical Instruments

The SPCE modification by 2,6-DHN electropolymerisation and all the other electrochemical experiments were performed with a potentiostat/galvanostat μAUTOLAB type II (Ecochemie, Amsterdam, The Netherlands). A boxed connector for screen-printed electrodes from Dropsens was used to connect the SPCEs. A single-line flow injection system was set up based on a four-channel peristaltic pump (Minipuls 3 from Gilson, Villiers-le-Bel, France) fitted with tygon tubing (1.12 mm id) for the propulsion of the carrier solution, an injection valve with 100 μL sample loop (model 7725i from Rheodyne, Cotati, CA, USA) and a flow-cell for screen printed electrodes (model DRP-FLWCL from Dropsens).

### 2.3. PON Synthesis

Peroxynitrite was synthesized from hydrogen peroxide and sodium nitrite according to a slightly modified method [39]. Briefly, in a 50 mL beaker containing 10 mL of 0.6 M H_2_O_2_ prepared in 0.7 M HCl kept on ice were rapidly added 10 mL of 0.6 M NaNO_2_ under vigorous magnetic stirring, followed 1 s later by 10 mL of ice cold 3 M NaOH to quench the reaction. Excess hydrogen peroxide was removed by adding 2 g of MnO_2_ flakes. MnO_2_ was removed by filtration and the obtained alkaline stock solution was aliquoted and kept in the dark at −20 °C. The peroxynitrite concentration of the stock solution was determined prior to each experiment by reading the absorbance at 302 nm (ε = 1670 L mol^−1^·cm^−1^).

### 2.4. Preparation of Poly(2,6-DHN)/SPCE

Poly(2,6-DHN) was electropolymerized on the surface of the carbon working electrode by cyclic voltammetry. The monomer solution consisted of 2 mM 2,6-DHN prepared in phosphate buffer saline (PBS) pH 7.4. The SPCEs were washed with bidistilled water before use. 100 μL of monomer solution were placed on the SPCE in order to cover all three (working, reference and counter) electrodes and the potential was cycled from −0.2 V to +1.2 V with scan rates of 5, 10 and 20 mV/s. After modification, the poly(2,6-DHN)/SPCE sensors were rinsed with water and dried in air. When not in use the sensors were kept in dark, at room temperature.

### 2.5. Morphological Analysis (Scanning Electron Microscopy)

The morphological analysis of modified/unmodified carbon electrodes was carried out with scanning electron microscope (SEM) images. The observations of the sensors surfaces were performed with a FEI-QUANTA 200 at the ICECHIM laboratory (Bucharest, Romania). The SEM images were taken at an accelerating voltage of 25–30 kV using a gaseous secondary electron detector (GSED). The micrographs of the samples were investigated at different magnifications to identify changes on the surface between modified and unmodified carbon electrodes. For each sensor, 4–7 micrographs were performed and the relevant images are presented.

### 2.6. Fourier Transform Infrared Analysis

Fourier transform infrared analysis (FTIR) was applied to evaluate the poly(2,6-DHN) formation on the carbon electrode. The FTIR spectra were acquired using a Tensor 37 FT-IR spectrometer (Bruker, Billerica, MA, USA) equipped with a Golden Gate ATR. All samples were recorded from 4000 to 400 cm^−1^.

### 2.7. Analytical Procedure

PON determination was performed by Flow Injection Analysis (FIA) amperometry. PON samples with a volume of 100 μL were injected via the injection valve in a carrier consisting of PBS with pH 9.0, propelled by the peristaltic pump with a flow rate of 0.38 mL/min. Potentials such as 0, +50, +75 and +100 mV were applied to the poly(2,6-DHN)/SPCE sensor introduced in the flow cell. Each sample was injected in triplicate.

### 2.8. Meat Extracts Analysis

The meat extract was prepared according to an adapted procedure used previously for nitrite determination [40]. 2.5 g of homogenized meat (veal under the age of 2) achieved from a local store was mixed with 25 mL of cold 0.1 M PBS pH 9.0. The mixture was vigorously stirred for 30 min at room temperature and then filtered through paper filter with large pores. Samples of meat extract diluted 10 times with PBS pH 9.0 and spiked with PON at different concentrations were analysed using the optimised FIA system.

## 3. Results and Discussion

### 3.1. Electrodeposition of Poly(2,6-DHN)

**Figure 1:**
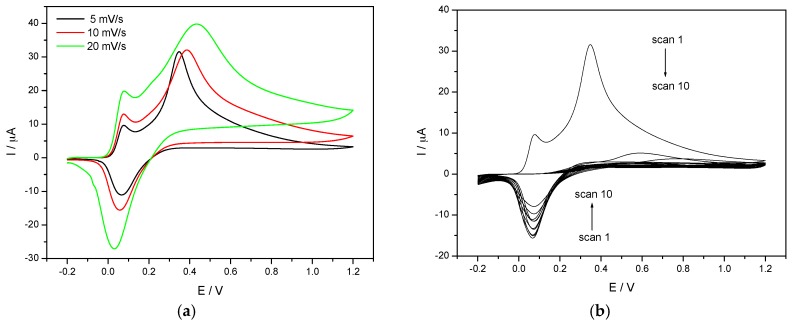
(**a**) First CVs of SPCE in 2 mM 2,6-DHN prepared in 0.1 M PBS pH 7.4 recorded at the scan rates 5, 10 and 20 mV·s^−1^; (**b**) 10 successive CVs of SPCE in 2 mM 2,6-DHN at scan rate 5 mV·s^−1^.

**Figure 2:**
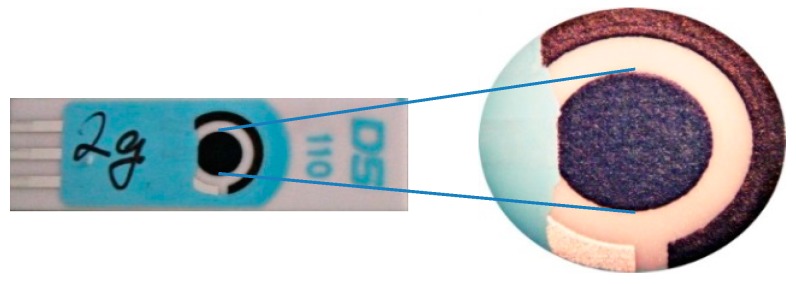
Aspect of the modified SPCE with poly(2,6-DHN).

The first cycle of the cyclic voltammograms performed for 2 mM 2,6-DHN solution at scan rates of 5, 10 and 20 mV·s^−1^, are characterized by a redox couple around 0 V and a broad oxidation peak around +0.4 V as it can be seen in Figure 1a. In Figure 1b one can observe that in the second and subsequent scans, the current of the oxidation peaks sharply decreased, indicating the polymerization process and the film formation. After completing 10 cycles of CV, the resulted poly(2,6-DHN)/SPCE electrodes were washed and dried in air. A dark blue film was formed on the carbon working electrode, as shown in Figure 2.

### 3.2. Characterization of Poly(2,6-DHN)/SPCE Sensors by SEM and FTIR Analysis

**Figure 3:**
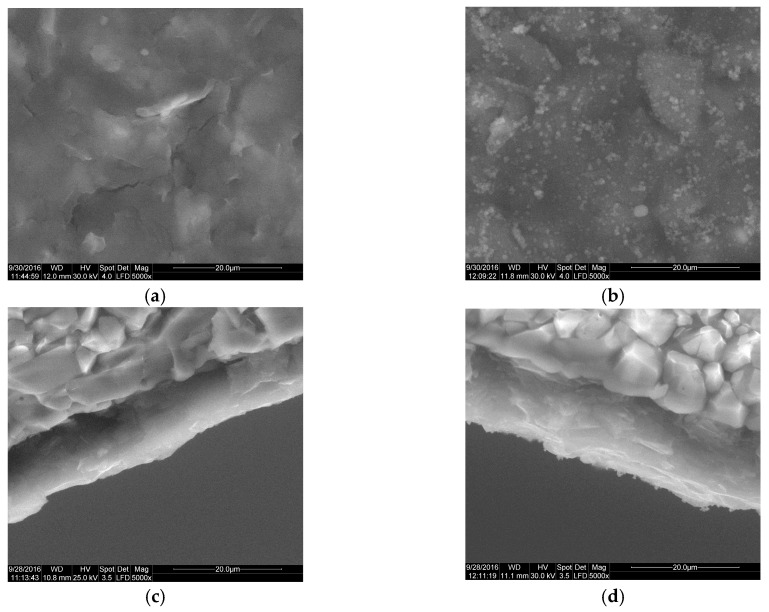
Frontal (**a**,**b**) and transversal (**c**,**d**) SEM images of the unmodified (**a**,**c**) and modified (**b**,**d**) SPCE.

The surface morphology of poly(2,6-DHN) film on the SPCE was characterized by SEM comparing with that of the unmodified SPCE. Figure 3 shows that following the electrodeposition process, the rough carbon surface of the working electrode was covered by a thin and reasonably homogeneous layer of poly(2,6-DHN). The mini-cracks visible on the unmodified carbon electrode are filled and covered with the polymeric film. The micro-particles present on the surface of the modified SPCEs may be phosphate salts entrapped in the polymer film during the electrodeposition.

**Figure 4:**
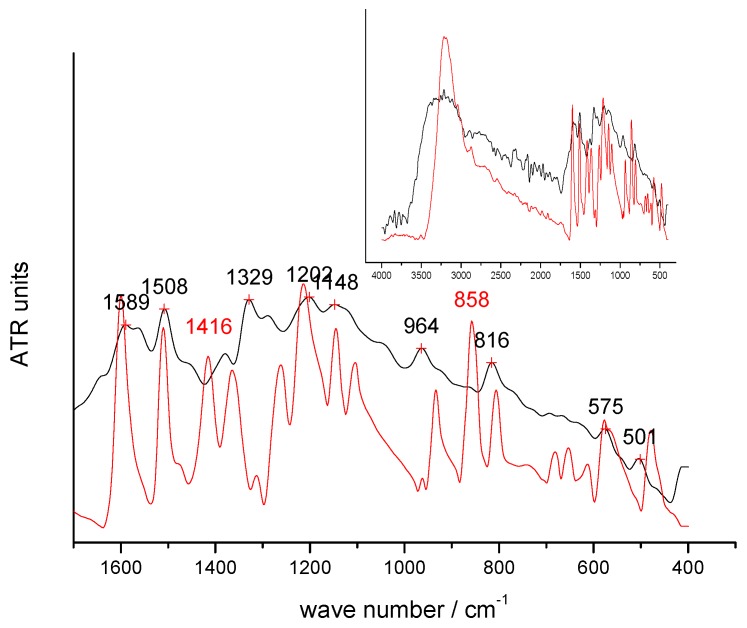
FTIR-ATR spectra for monomer 2,6-DHN (red line) and poly(2,6-DHN)/SPCE (black line).

The structure of the poly(2,6-DHN) film electrodeposited on the SPCE was investigated also by FTIR-ATR analysis. In Figure 4 the FTIR-ATR spectra of the pure monomer 2,6-DHN and of the poly(2,6-DHN) are presented.

The strong and broad peak at around 3200 cm^−1^ observed in the spectrum of 2,6-DHN (Figure 4 inset) characteristic to the O–H stretching vibration, became broader and shifted to 3400 cm^−1^ in the spectrum of the poly(2,6-DHN film). This band, together with the band at 1329 cm^−1^, can be assigned to O–H deformation vibration. The band at 1202 cm^−1^ can be ascribed to the C–OH stretching vibration. These results indicate that the O–H groups still exist in the poly(2,6-DHN) structure, and the hydroxyl groups did not represent the polymerization sites. Also, the peaks at 1589 cm^−1^ and 1508 cm^−1^ characteristic to C–C stretching suggest that the irreversible anodic reaction probably proceeds via C-C bond forming polymerization. A similar behavior was reported for the oxidative coupling polymerization of 2,6-DHN in basic water, when amines were used as base [41].

**Scheme 1:**
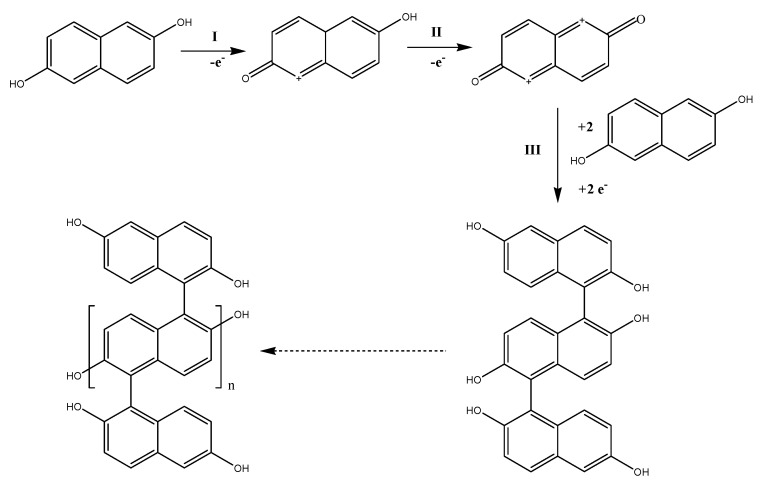
Suggested electropolymerisation mechanism and structure for poly(2,6-DHN).

The suggested structure of the electropolymerized poly(2,6-DHN) and the proposed mechanism for its electrosynthesis are shown in Scheme 1.

In the first polymerization cycle, the two oxidation peaks are attributed to reactions I and II, and the reduction peak is attributed to reaction III when a trimer is formed. In the next cycle, the available sites for coupling decrease and only one small oxidation peak shifted to more positive potential is observed. With increasing the number of the cycling scans the oxidation peak almost disappears and the number of 2,6-DHN units in the polymer structure increases.

### 3.3. Electrochemical Characterization of the Poly(2,6-DHN)/SPCE Sensors

**Figure 5:**
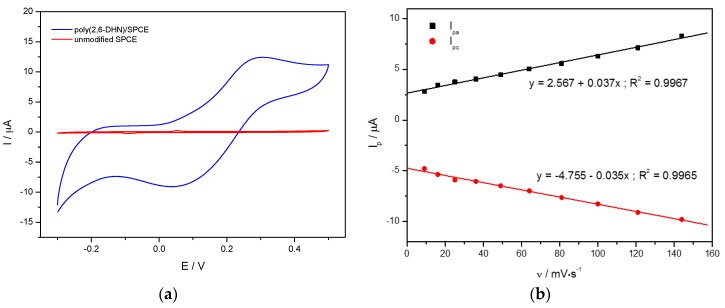
(**a**) CVs for poly(2,6-DHN)/SPCE and unmodified SPCE in 0.1 M PBS pH 9.0 . Scan rate 100 mV·s^−1^; potential range: −0.3 V to +0.5 V; sensor modified by electropolymerization of 2,6-DHN with the scan rate = 5 mV·s^−1^. (**b**) Dependence of the anodic and cathodic peak currents (I_p_) on scan rate (ν) for poly(2,6-DHN)/SPCE in 0.1 M PBS pH 9.0. Scan rates: 9–144 mV·s^−1^; sensor modified by electropolymerization of 2,6-DHN with the scan rate = 5 mV·s^−1^.

Figure 5a shows the voltammetric behavior of the unmodified SPCE and of the poly(2,6-DHN)/SPCE in PBS pH 9.0. No redox peaks were observed for the unmodified SPCE, meanwhile a pair of reversible peaks may be observed for the poly(2,6-DHN)/SPCE. The anodic and cathodic peaks are situated at ~+270 mV and ~+97 mV, respectively. We attribute the anodic peak to the oxidation of the 2,6-DHN units present in the polymer to naphthalene-2,6-dione and the cathodic peak to the reduction back to 2,6-DHN. The dependence of the peak current (I_p_) on the scan rate (ν) is presented in Figure 5b. For scan rates below 150 mV·s^−1^, there is a linear relation between I_p_ and ν characteristic for a surface-immobilized couple. Above 200 mV s^−1^ the I_pa_ reaches a plateau and above 300 mV·s^−1^ it starts to decrease (data not shown). It seems that at high scan rates the available time window is not large enough to allow the polymer electrooxidation. For the range of the scan rates studied the peak potentials are almost independent on the scan rate and the redox reaction is apparently reversible. A similar behavior for scan rates above 150 mV·s^−1^ was reported for a catechin film electrodeposited on glassy carbon [42].

**Figure 6:**
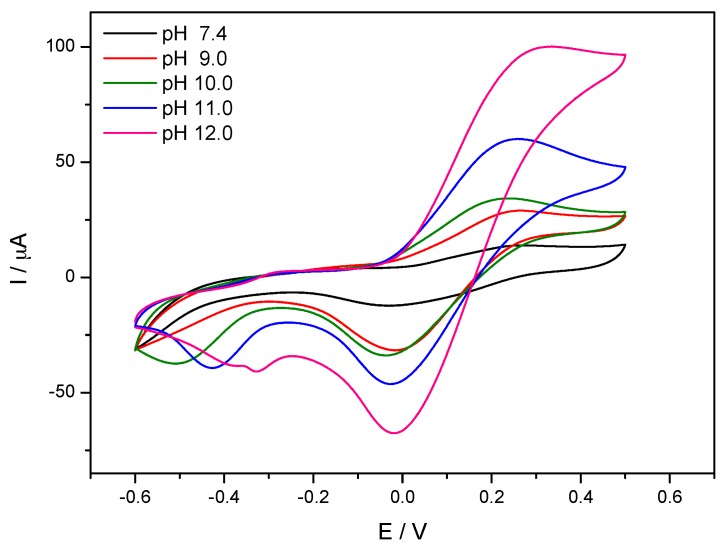
The influence of electrolyte solution pH on CVs of poly(2,6-DHN)/SPCE. PBS pH: 7.4, 9.0, 9.4, 10.0, 11.0, 12.0. Potential range: −0.5 V to +0.5 V; scan rate: 100 mv·s^−1^; sensor modified by electropolymerization of 2,6-DHN with the scan rate of 20 mV·s^−1^.

Taking into consideration the stability of PON in high alkaline solution, the poly(2,6-DHN)/ SPCEs were characterized in 0.1 M PBS with different pH between 7.4 and 12, by cycling the potential from −0.6 V to +0.5 V, with the scan rate 100 mV·s^−1^. Greatly enhanced peaks, both anodic and cathodic, with the pH increasing were observed (Figure 6). Also, it was observed that the potential of the peaks is not influenced by pH, indicating that the hydrogen ions are not involved in the electrode reaction. The increasing of the peak currents with pH suggests that the electrooxidation of the 2,6-DHN units to naphthalene-2,6-dione involves hydroxyl ions. However, it was observed that the high pH is not favorable for the film stability and integrity. As a compromise, pH 9.0 electrolyte solution was selected for further experiments.

**Figure 7:**
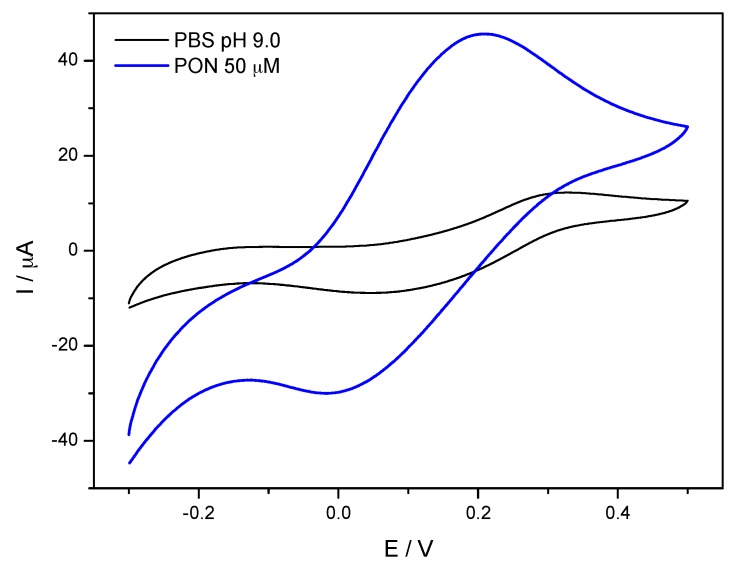
Cyclic voltammograms of poly(2,6-DHN)/SPCE in 0.1 M PBS pH 9.0, in the presence (bue line) and absence (black line) of 50 μM PON. Potential range: −0.3 V to +0.5 V; scan rate: 100 mv·s^−1^.

The electrocatalytic behavior of the poly(2,6-DHN)/SPCEs was also evaluated by cyclic voltammetry in the presence and absence of 50 μM PON in PBS pH 9.0. The response of the sensor prepared with a scan rate of 5 mV·s^−1^, presented in Figure 7, shows a significant increase of the anodic and cathodic peaks characteristic to the poly(2,6-DHN) film in the presence of PON. PON induced potentials shift of both anodic and cathodic peak to lower values. One may also observe that in the presence of PON the anodic peak is higher than the cathodic peak. These results indicate that the poly(2,6-DHN)/SPCE sensor has electrocatalytic activity towards the PON oxidation. All poly(2,6-DHN)/SPCE sensors showed similar responses, independent on the scanning rate used for electropolymerization of 2,6-DHN.

**Figure 8:**
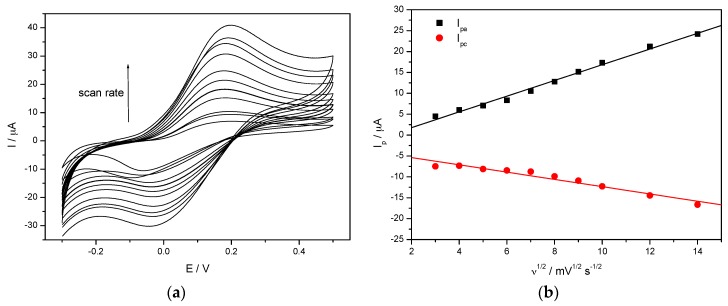
(**a**) CVs of poly(2,6-DHN)/SPCE in 0.1 M PBS pH 9.0 containing 50 μM PON at scan rates from 9 to 250 mV·s^−1^. Potential range: −0.3 V to +0.5 V; sensor modified by electropolymerization of 2,6-DHN with the scan rate of 5 mV·s^−1^. (**b**) Plot of the anodic and cathodic peak currents vs. square root of scan rate (ν^1/2^)

In order to get kinetic information the influence of the scan rate was tested for 50 μM PON in 0.1 M PBS pH 9.0. Figure 8 shows that both the anodic and cathodic peaks increased linearly with the square root of the scan rate, suggesting a typical behavior of a diffusion controlled reaction [43].

### 3.4. Amperometric Detection of PON

**Figure 9:**
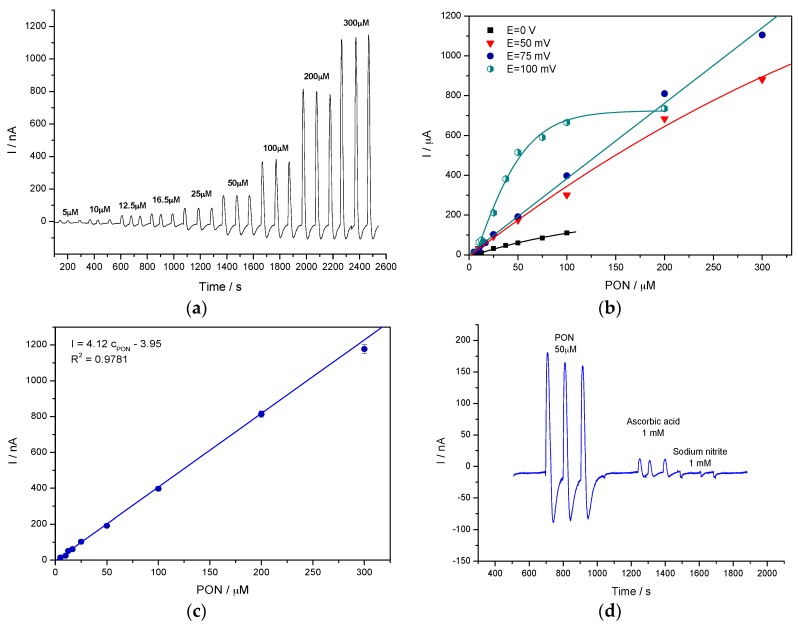
(**a**) FIA amperogram for different PON concentration solutions. E = +75 mV; flow rate = 0.38 mL·min^−1^; (**b**) Dependence of FIA height on the applied potential for different concentration of PON; flow rate = 0.38 mL·min^−1^; (**c**) Calibration graph for PON determination by FIA amperometry in optimized conditions; E = +75 mV; flow rate = 0.38 mL·min^−1^; (**d**) FIA signals for PON 50 μM, ascorbic acid 1 mM and sodium nitrite 0.1 M; E = +75 mV; flow rate = 0.38 mL·min^−1^.

The Flow Injection Analysis (FIA) technique was used for the amperometric detection of PON using the developed poly(2,6-DHN)/SPCE sensor. A very simple single-line FIA system based on injection of 100 μL sample in a flow of PBS pH 9.0 pumped with an optimized flow rate of 0.38 mL·min^−1^ was used. When the samples reach the electroactive surface of the sensor which is inserted in a special flow cell for screen printed electrodes characteristic FIA peaks are recorded such as shown in Figure 9a. The height of the FIA peaks is proportional to the concentration of PON solution injected. The flow rate is important to be optimized in FIA in order to achieve the highest ratio between the FIA signal and background noise. Flow rates between 0.2 and 0.5 mL·min^−1^ were investigated and the optimum was set up at 0.38 mL·min^−1^ (data not shown).

In amperometry the choice of the optimum potential applied at the working electrode represents the key parameter in achieving the lowest limit of detection (LOD) and the higher sensitivity. The influence of the applied potential on the height of the FIA signals for different PON concentrations as well as on the background noise was investigated for E of 0, 50, 75 and 100 mV vs. Ag/AgCl pseudoreference. In Figure 9b the plots representing the dependence of FIA peaks height on PON concentration for the investigated applied potentials are shown. For the potential of 100 mV the highest sensitivity (11.62 nA·μM^−1^) was recorded, but the linear range is limited from 1 μM to 50 μM, and the background is relatively noisy. At 0 V applied potential the lowest sensitivity was achieved (1.13 nA·μM^−1^) and for potentials of 50 and 75 mV the sensitivity was similar for the PON concentration range up to 100 μM. The optimum applied potential was found to be 75 mV vs. Ag/AgCl pseudoreference due to the broadest concentration range of PON (2–300 μM) and a sensitivity of 4.12 nA·μM^−1^ (Figure 9c). The formula LOD = 3 σ_b_/m was used to calculate the limit of detection, were σ_b_ represents the standard deviation of the background and m is the slope of the calibration graph. For the optimum applied potential of 75 mV a LOD of 0.2 μM PON was achieved. Similarly, a LQD (limit of quantification) of 0.7 μM was calculated according to the formula LQD = 10 σ_b_/m.

### 3.5. Selectivity, Reproducibility and Stability of the Poly(2,6-DHN)/SPCE

In order to test the applicability of the developed sensor for PON detection in real samples, some possibly coexisting interferents in meat extracts, such as ascorbic acid and nitrite, were investigated. Figure 9d shows the FIA responses for injection of 1 mM scorbic and 1 mM sodium nitrite solutions compare with that recorded for injection of 50 μM PON.

It is evident that nitrite does not interfere in PON determination and for a 20 times higher concentrated solution of ascorbic acid than that of PON, the interference was low (around 7%). The near zero operation voltage of the poly(2,6-DHN)/SPCE succeeds to eliminate the interference of these two important compounds which create major difficulties in PON detection with sensors operating at high potentials. The intra-electrode reproducibility (for the same electrode) investigated for a PON concentration of 50 μM resulted in a relative standard deviation (RSD) of 2.3% for ten consecutive measurements. For the inter-electrode reproducibility study, the RSD determined for six different electrodes was 5.9 % confirming that the fabrication method of the poly(2,6-DHN)/SPCE is highly reproducible.

The operational stability of the developed sensor was examined by successive injections of 50 μM. It was observed that in the case of the sensor prepared by electropolymerization of 2,6-DHN with the scan rate of 20 mV·s^−1^, after 10 successive injections the signal decreased with 15% and the background noise increased. The optical and FTIR analysis revealed that the integrity of the poly(2,6-DHN) film was partially affected. Probably, the film electropolymerized at high scan rates does not provide the appropriate features to keep the film integrity in the flowing conditions required for FIA analysis. For the other two electropolymerization scan rates (5 and 10 mV·s^−1^), in operational conditions, the amperometric response for PON 50 μM retained about 90% of its initial value after 30 injections. Also, the electrodes stored in air when not in use were able to retain 91%–93% of their original responses after two weeks storage, indicating good stability.

### 3.6. PON Recovery in Meat Extract

**Figure 10:**
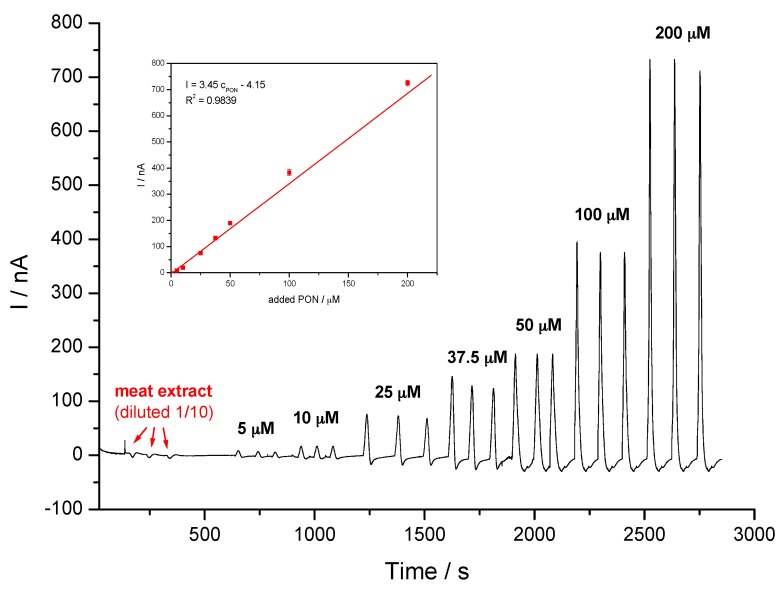
FIA amperogram for PON added in meat extract (diluted 1/10 with PBS pH 9.0). Inside: calibration graph for PON in meat extract. E = +75 mV; flow rate = 0.38 mL·min^−1^

For evaluation of the applicability of the developed sensor in real samples, a calibration graph was constructed based on the FIA peaks recorded for samples consisting of meat extract (diluted 10 times with PBS pH.9) spiked with certain amounts of PON in order to reach concentrations between 5 and 200 μM (Figure 10). The samples were injected 15 s after spiking. In Figure 10 it can be observed that the meat extract itself without added PON gave a very small reverse FIA peak. The calibration graph for PON added in meat extract showed a good linearity for the range 5–200 μM. The sensitivity of 3.45 nA·μM^−1^ represents ~84% from the sensitivity recorded for PON determination in PBS pH 9.0, meaning that the meat matrix has a reduced influence on PON determination with the poly(2,6-DHN)/SPCE sensor. The PON recovery in meat extract is 96%–99% for added PON concentration below 50 μM and 93%–96% for higher concentration.

In Table 1 a comparison between the analytical features of the sensor developed in this work with other similar sensors reported in the literature is presented.

## 4. Conclusions

The electropolymerization of 2,6-dihydroxynapthalene (2,6-DHN) on carbon electrode (screen printed) is reported for the first time. The poly(2,6-DHN)/SPCE demonstrates excellent electrocatalytic activity for PON oxidation in alkaline solutions at very low potential (0–100 mV vs. Ag/AgCl pseudoreference). An amperometric FIA system based on the developed poly(2,6-DHN)/SPCE sensor was optimized for PON measurements. The optimized sensor and FIA system exhibited good selectivity, sensitivity, reproducibility and stability for PON determination, as well as low LOD (0.2 μM PON). All these analytical features of the new carbon electrode modified with poly(2,6-DHN) represent a good premise for a future work related to the integration of this sensor in a smart label for detecting the meat freshness.

## Figures and Tables

**Table 1:** Comparison between the analytical features of different PON sensors.

Mediator/Electrode	Electroanalytical Method	Linear Range (μM)	LOD (μM)	Sample	Reference
Manganese tetraamino-phthalocyanine/Pt microelectrode	Amperometry; Reduction—E = −0.5 V	16.6–115	5.0	-	[26]
Manganese-[poly2,5-di-(2-thienyl)-1*H*-pyrrole)-1-(*p*-benzoic acid)]/Pt microelectrode	Amperometry; Reduction—E = 0.2 V	0.02–0.05	0.0019	Blood plasma; Cell culture	[27]
Cyanocobalamin/glassy carbon electrode	Differential pulse voltammetry	20–300	0.1	Human serum	[30]
hemin–PEDOT/carbon fiber microelectrode	Amperometry; Oxidation—E = 0.75 V	1–400	0.2	-	[32]
Reduced graphene oxide/glassy carbon electrode	Amperometry; Oxidation—E = 1.1V	0.005–0.016	0.005	-	[34]
Poly(2,6-DHN)/screen printed carbon electrode	Amperometry; Oxidation—E = 0.075V	2–300	0.2	Meat extract	This work
